# Application of Fuzzy Logic in Oral Cancer Risk Assessment

**Published:** 2017-05

**Authors:** Ioana SCROBOTĂ, Grigore BĂCIUȚ, Adriana Gabriela FILIP, Bianca TODOR, Florin BLAGA, Mihaela Felicia BĂCIUȚ

**Affiliations:** 1. Dept. of Cranio-Maxillofacial Surgery and Radiology, Iuliu Hațieganu University of Medicine and Pharmacy, Cluj Napoca, Romania; 2. Dept. of Dental Medicine, Faculty of Medicine and Pharmacy, University of Oradea, Oradea, Romania; 3. Dept. of Radiobiology and Tumour Biology, ”Prof. Dr. I. Chiricuță” Oncology Institute, Cluj-Napoca, România; 4. Dept. of Physiology, Iuliu Hațieganu University of Medicine and Pharmacy, Cluj-Napoca, Romania; 5. Dept. of Industrial Engineering, Faculty of Managerial and Technological Engineering, University of Oradea, Oradea, Romania; 6. Dept. of Implantology and Maxillofacial Surgery, Iuliu Hațieganu University of Medicine and Pharmacy, Cluj Napoca, Romania

**Keywords:** Oral potentially malignant disorders, Malondialdehyde, Proton donors, Cancer risk, Fuzzy logic

## Abstract

**Background:**

The mapping of the malignization mechanism is still incomplete, but oxidative stress is strongly correlated to carcinogenesis. In our research, using fuzzy logic, we aimed to estimate the oxidative stress related-cancerization risk of the oral potentially malignant disorders.

**Methods:**

Serum from 16 patients diagnosed (clinical and histopathological) with oral potentially malignant disorders (Dept. of Cranio-Maxillofacial Surgery and Radiology, ”Iuliu Hațieganu” University of Medicine and Pharmacy, Cluj Napoca, Romania) was processed fluorometric for malondialdehyde and proton donors assays (Dept. of Physiology,”Iuliu Hațieganu” University of Medicine and Pharmacy, Cluj-Napoca, Romania). The values were used as inputs, they were associated linguistic terms using MIN-MAX method and 25 IF-THEN inference rules were generated to estimate the output value, the cancerization risk appreciated on a scale from 1 to 10 - IF malondialdehyde is very high and donors protons are very low THEN the cancer risk is reaching the maximum value (Dept. of Industrial Engineering, Faculty of Managerial and Technological Engineering, University of Oradea, Oradea, Romania) (2012–2014).

**Results:**

We estimated the cancerization risk of the oral potentially malignant disorders by implementing the multi-criteria decision support system based on serum malondialdehyde and proton donors’ values. The risk was estimated as a concrete numerical value on a scale from 1 to 10 depending on the input numerical/linguistic value.

**Conclusion:**

The multi-criteria decision support system proposed by us, integrated into a more complex computerized decision support system, could be used as an important aid in oral cancer screening and establish future medical decision in oral potentially malignant disorders.

## Introduction

Oral cancer is a worldwide spread cancer and occupies the 7^th^ position in EU, with a high incidence in Eastern Europe (1). In the last decades, carcinogenesis was an intensively studied process but there is no amelioration noticed in its prognostic (2). More than 80% of oral cancers are squamous cell carcinomas developed from oral potentially malignant disorders (3). Therefore, their early diagnosis, adequate screening, and estimation of the cancerization risk could considerably improve the management of this disease. Any decision making requires large quantities of information. During carcinogenesis, following the exposure to carcinogens, an entire sequence of genetic, epigenetic and metabolic modifications occurs and numerous factors are implicated (4). The disease may manifest differently with different intensities in different patients. Some data, most of them provided by the patients, involve uncertainties. For a clinician it is difficult to interpret all this volume of information, therefore, data should be automated and used effectively. Conventional statistical methods were applied but they take a lot of time and they are not suitable for all cases (5).

An appealing alternative to the existing prediction tools is artificial intelligent prediction. This method is based on human-like learning ability in pattern recognition and generalization known as machine learning. Researchers designed and used machine-learning algorithms proved to be of great value. Various numerous studies propose the use of artificial intelligence in medicine, with a particular emphasis on cancer. The majority of them identify, classify, detect, or distinguish tumors and only a few predict or prognoses cancer (6–8).

The present paper proposes the use of fuzzy logic – a machine-learning algorithm - to assess cancer risk in screening oral potentially malignant disorders.

Fuzzy logic is a superset of the conventional logic coined in 1965 and used in mathematics and Systems Theory under the name of “fuzzy” set. Relative to the classical conception of the set and the set element in which an existence either is an element of the set or is not, a fuzzy set represents a completely new approach to these ones. More precisely, between the element membership and element non-membership, there is an entire range of transitory, continuous situations, characterized by values indicating the degrees of membership (9, 10). Fuzzy logic in its simplest terms expands the dichotomy of true or not true to include a range of degrees of truth answers in between. Introducing partial truths, fuzzy logic is more appropriate in medicine where diagnosis implies complex data involving several levels of uncertainty and imprecision (11, 12).

An important advantage that can stand alone in justifying the use of fuzzy logic in medicine is the ability of this machine algorithm to introduce into the process of decision linguistic terms, easier for human users to understand and communicate with (13, 14).

## Materials and Methods

A number of 16 patients were diagnosed with oral potentially malignant disorders based on clinical data and histopathological exam (Dept. of Cranio-Maxillofacial Surgery and Radiology, ”Iuliu Hațieganu” University of Medicine and Pharmacy, Cluj Napoca, Romania).

An amount of 5 ml venous blood was sampled and immediately centrifuged at 3500 rotations/ min. The resulting serum was frozen and kept until processed to determine two biochemical parameters. Serum total malondialdehyde (MDA) (15) and serum proton donor capacity (DONORS_PROTONS) (16) were assayed (Dept. of Physiology, “Iuliu Hațieganu” University of Medicine and Pharmacy, Cluj-Napoca, Romania).

**Fig. 1:**
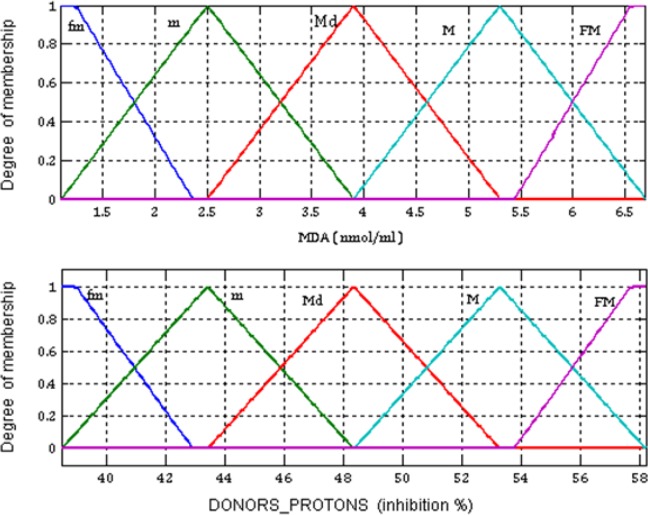
Input values in the multi-criteria decision support system

**Fig. 2:**
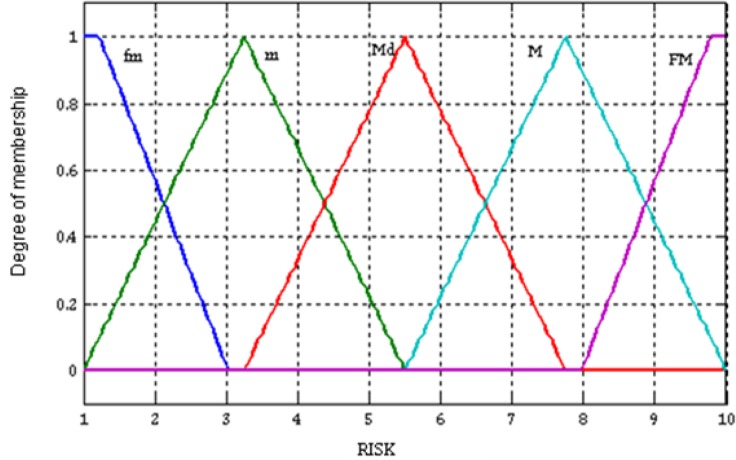
Output values in the multi-criteria decision support system

We used fuzzy logic to interpret the values in the input data and, based on a set of rules, to assign values to the output. The following steps were taken in order to implement the multi-criteria decision support system used for cancer risk determination (Dept. of Industrial Engineering, Faculty of Managerial and Technological Engineering, University of Oradea, Oradea, Romania) (2012–2014):

- Defining criteria (input data in decision-making)
  1. MDA;
  2. DONORS_PROTONS.
    MDA=[1.1000; 6.7000]    [nmol/ml]
- Defining the variation fields of input values
  DONORS_PROTONS=[38.5000; 58.2000]    [inhibition%]
  The intervals were determined by the minimum and maximum values of the serum parameters (MDA and DONORS_PROTONS, respectively) in patients presenting oral potentially malignant disorders.
  We introduced linguistic terms (LT) into the process of decision and built membership functions to map numeric data of the inputs to the LT:
- Defining linguistic terms (LT) associated with each input value and
  $$\begin{array}{l} \mathrm{MDA}:\;LT_{\mathrm{MDA}}=\{fm,m,\mathrm{Md},M,\mathrm{Fm}\}\;\;\text{and}\\ \mathrm{DONORS}\_\mathrm{PROTONS}:\;\;LT_{\mathrm{DONORS}\_\mathrm{PROTONS}}=\{\mathrm{fm},m,\mathrm{Md},M,\mathrm{Fm}\}, \end{array}$$
  respectively
  Where: FM- very small; m- small, Md- medium, M- high, FM- very high.
  Different membership functions were used to convert the numerical inputs to the LT: FM- trapezoidal; m, Md, M- triangular; and FM- trapezoidal (Fig. 1).
- Defining the output values in the multi-
  $$\mathrm{RISK}:D_{\mathrm{RISK}}=[1;10]$$
  criteria decision support system - the *ILLNESS RISK.*
  $$\mathrm{RISK}:\;LT_{\mathrm{RISK}}=\{\mathrm{fm},m,\mathrm{Md},M,\mathrm{Fm}\}$$
- Defining the variation fields of input values:
- Defining the LT associated with each output value:
  where: *FM- very small; m: small, Md- medium, M- high, FM: very high.*
  Different membership functions were used to convert the numerical outputs to the LT: fm- trapezoidal; m, Md, M- triangular; and FM- trapezoidal (Fig. 2).

**Fig. 3:**
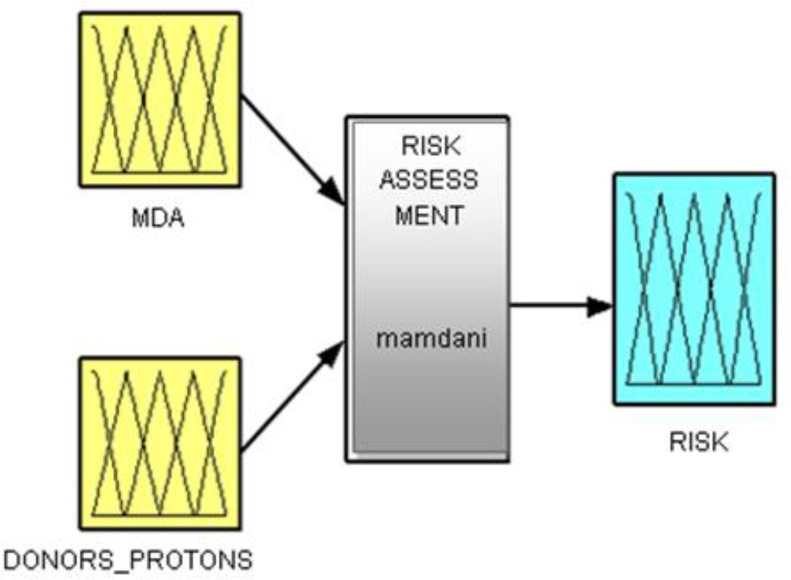
Decision-making system implemented in Fuzzy Logic Toolbox / Matlab®

By using *MIN-MAX method,* we generated 25 inference rules to match the various LT and obtain the outputs. We applied the IF-THEN rule - IF MDA is increased and DONORS_PROTONS are decreased THEN the RISK of developing cancer is elevated (Fig. 3 and Table 1).

**Table 1:** Inference rules based on the IF-THEN rule – matching linguistic terms to obtain outputs

	**IF**		**THEN**
	**MDA**	**DONORS_PROTONS**	**RISK**
1.	fm	Fm	Md
2.	fm	M	Md
3.	fm	Md	m
4.	fm	M	fm
5.	fm	FM	fm
6.	m	Fm	Md
7.	m	M	Md
8.	m	Md	m
9.	m	M	m
10.	m	FM	fm
11.	Md	Fm	M
12.	Md	M	Md
13.	Md	Md	Md
14.	Md	M	m
15.	Md	FM	m
16.	M	Fm	M
17.	M	M	M
18.	M	Md	Md
19.	M	M	Md
20.	M	FM	m
21.	FM	Fm	FM
22.	FM	M	FM
23.	FM	Md	M
24.	FM	M	Md
25.	FM	FM	Md

This study followed the ethical standards of the Helsinki Declaration, as revised in 2013 and has been approved by the Ethical Committee of Iuliu Hațieganu University of Medicine and Pharmacy. All of the patients gave their informed consent prior any investigation was conducted.

## Results

The implementation of the multi-criteria decision support system resulted in estimation of the output numerical value - ILLNESS RISK by introducing in the system input numerical values - MDA and DONORS_PROTONS. The highest MDA value associated with the lowest DONORS_PROTONS value in the variation fields corresponds to the highest value of ILLNESS RISK. The lowest MDA value associated with the highest DONORS_PROTONS value corresponds to the lowest value of ILLNESS RISK (Table 2). For each value in the variation fields, the system generates a risk value (Table 2). For an MDA value of 6.5880 nmol/ml considered very high (Fig. 1) and a DONORS_PROTONS value of 38.8950 inhibition % considered very low (Fig. 1) the risk of developing cancer has an 8.9813 value on a scale from 1 to 10 – the ILLNESS RISK is very high (Fig. 2, Table 2).

**Table 2:** Values resulting from the procedure implementation

	**MDA**	**DONORS_PROTONS**	**RISK**
1	6.7000	38.5000	9.3440
2	6.6440	38.6975	9.1448
3	6.5880	38.8950	8.9813
4	6.5320	39.0925	8.8406
5	6.4760	39.2900	8.7140
…	…	…	…
44	4.2920	46.9925	6.2051
45	4.2360	47.1900	6.1213
46	4.1800	47.3875	6.0331
47	4.1240	47.5850	5.9395
48	4.0680	47.7825	5.8392
…	…	…	…
97	1.3240	57.4600	2.2594
98	1.2680	57.6575	2.1276
99	1.2120	57.8550	1.9800
100	1.1560	58.0525	1.8076
101	1.1000	58.2500	1.6560

**Fig. 4:**
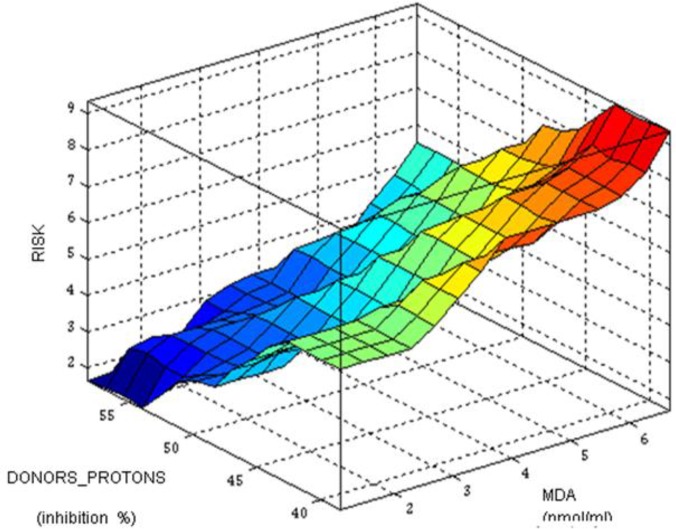
Variation surface of the RISK

The dependence of the output value of the input values generated a variation surface of the RISK presented in Fig. 4. The risk increases from blue color to red color – the elevated MDA and the lowered DONORS_PROTONS.

## Discussion

In the present study, we propose the use of fuzzy logic to assess the oxidative stress-related oral cancer risk.

We elaborated a multi-criteria decision support system that can utilize data introduced by the user (inputs) to generate an answer (output).

The exact mechanism underlying the malignant progression of oral cancer is still unclear but a strong relationship exists between cancer and oxidative stress (17–21). When the balance between oxidants and antioxidants is altered, oxidative stress is installed and a certain degradation of a cell, tissue or organ is produced (22). MDA is a final product of lipid peroxidation, one of the most frequent and important oxidative processes. Serum MDA is a reliable indicator of the whole body oxidative status and it was found increased in numerous pathologies including oral cancer (23). DONORS_PROTONS are an expression of the antioxidant activity of the organism in the effort of counteracting oxidative stress and they were found decreased in several diseases including oral cancer (24). Therefore, we used oxidative stress indicators - MDA and DONORS_PROTONS as inputs but, based on the medical experience of the user, different couples of input values could be used.

In order to provide a natural way of communicating we incorporated LT in the system (14). To attribute LT to the numerical inputs and output we used membership functions. We applied the most common functions used, functions formed using straight lines, as the triangular membership function and trapezoidal membership function (25). There are several other more unusual membership functions that may have application in fuzzy systems (26) and we plan to evaluate their influence on the accuracy of prediction as a future research direction.

Human logic is based on IF-THEN rules. Fuzzy logic aims to translate these IF-THEN rules to machines in order to augment their efficiency (27). The rules to map LT of inputs and output were IF-THEN rules: if MDA is increased and DONORS_PROTONS are decreased then the cancer RISK is increased. We established the rules because increased serum oxidative stress and decreased serum total antioxidant capacity were measured in oral cancer patients versus those presenting oral potentially malignant disorders (28–30).

In several researches machine learning was applied as a tool in cancer management [6]. By now, fuzzy sets were used to predict cervical lymph node metastasis in carcinoma of the tongue (31), for the prognosis of nasopharyngeal carcinoma (32), outcome prediction in esophageal cancer (33), for the prediction of oral cancer susceptibility (34). Cancer prediction or prognosis is different from cancer detection and diagnosis and it refers to the estimation of the susceptibility of developing the disease and the prediction of its recurrence and survivability (6, 35). This is the first study that proposes the use of fuzzy logic in oral cancer susceptibility/risk assessment of oral potentially malignant disorders using as inputs oxidative stress parameters.

The clinical ability to predict oral potentially malignant disorders cancerization is limited and the common histopathological exam offers reduced provisional value because the diagnosis is subjective; not all cases of potentially malignant disorders or even dysplasias necessarily evolve into cancer, some even being able to regress; carcinoma can also occur in lesions not presented any previous dysplasia. Specific biomarkers such as oncogenes, tumor suppressor gene mutations, cell cycle proteins, or DNA transcription factors were taken into consideration, however, up to this point/ it has been difficult to predict which oral potentially malign disorder will evolve to cancer (36–38).

Fuzzy logic is an appropriate modeling tool for prediction because it could reach general solutions by using limited data, even uncertain verbal information characteristic to human logic (39).

As important future research directions, we plan to validate this method by experimental and clinical studies; evaluate the influence of the transfer function on the accuracy of prediction; establish the illness risk based on different couples of input values, and generalize the method for *n* input values; develop a user-friendly (interface-user) integrated computer system for cancer risk assessment.

## Conclusion

Artificial intelligence could improve the methods used in predicting cancerization of oral potentially malignant disorders. Fuzzy logic, in particular, has the advantage of allowing the use of ambiguous values as input data unreliable to other methods; and facilitating a correspondence between the numerical values of the disease parameters and linguistic terms, easier to process by the user.

This multi-criteria decision support system proposed by us can be integrated into a more complex computerized decision support system. A user-friendly (interface-user) integrated computer system that uses first and/or only minimum invasive sampling (serum or saliva) markers to estimate the cancer risk of oral potentially malignant disorders can be, in our opinion, an important clinicians aid in screening and establishing future medical decision.

## Ethical considerations

Ethical issues (Including plagiarism, informed consent, misconduct, data fabrication and/or falsification, double publication and/or submission, redundancy, etc.) have been completely observed by the authors.
